# Historical and Critical View of the Small-Pox Epidemics, &c.

**Published:** 1839-01

**Authors:** 


### Art. X.

1. *Historisch-kritisch Darstellung der Pockenseuchen, des gesammten Impf-und Revaccinationswesens im Königreiche Württemberg, innerhalb die fünf Jahre Juli,* 1831, *bis Juni,* 1836. Von Professor Dr. Franz Heim, K. Württ. Regimentsarzte, &c. &c.—*Stuttgart,* 1838. 8vo. pp. 651.

*Historical and Critical View of the Small-pox Epidemics, and of the entire Vaccinations and Revaccinations in the Kingdom of Wirtemberg, from July,* 1831, *to June,* 1836, *inclusive.* By Professor Francis Heim, m.d. &c. &c.—*Stutgard,* 1838.

2. *Resultate der Revaccination in dem Königlich Würtembergischen Militär, in den Jahren* 1833, 1834, *und* 1835. Von Professor Heim. —*Ludwigsburg,* 1836. 8vo. pp. 100.

*Results of the Revaccination in the Royal Wirtemberg Army, in the Years* 1833, 1834, *and* 1835. By Prof. Heim.—*Ludwigsburg,* 1836.

The casual observations on the subject of revaccination which we felt ourselves called upon to make in our last Number, when reviewing the very interesting work of Dr. Baron, will have prepared our readers to expect a more detailed examination of this all-important question. The discussion of the rise and progress of vaccination is so intimately blended in every particular with the life of Jenner, and has been so judiciously made a prominent feature in the biography recently completed by Dr. Baron, that the objections formerly urged, and others more recently brought forward, against the practice of vaccine inoculation, as well as the facts and arguments by which these objections are met, must be fresh in the minds of all. Whether these objections are valid or not, whether they are partially correct or are to be considered as entirely without foundation, is however, unhappily, not at the present time a mere matter of speculation. We have recently and again been called upon to witness the ravages of small-pox amongst us: we have watched its progress at the bed-side, case after case, and have seen the sufferers fall a prey to all its loathsome horrors.

The debt of gratitude which mankind owes to Jenner is indeed beyond all estimation, and his name must ever have been enrolled among the noblest of the benefactors of the human race, even had the protection introduced by the practice of vaccination ceased with the existence of him who called it into being. We must not, however, permit ourselves to be dazzled or led astray by our respect for any individual, however exalted; but, guided by his bright example, let us rather seek, with the same unwearied perseverance and the same unabated energy, the object which he sought with so much simplicity of mind and singleness of purpose. It is not by shrinking from investigation that we shall ever arrive at the knowledge of truth; and we owe it alike to the memory of Jenner, to ourselves, and to those who look to us for advice and direction, to ascertain how far vaccination is entitled to our confidence as a protective from the ravages of small-pox; how far the failures which recent experience demonstrates to have become more frequent are to be attributed to imperfection in the agent itself or in the mode in which it is applied; to modifications which time may have wrought in its very essence, or to carelessness, ignorance, or neglect in its employment.

That complete protection has been afforded to multitudes is undeniable; that comparative protection is afforded to a very large majority of those subjected to the vaccine influence is equally clear; but that numerous partial failures have occurred, and some (we must with pain admit) in which the operation has been absolutely of no avail, cannot now be disputed. This, however, only affords the stronger grounds for calm and close enquiry into the causes of failure; and the benefit unquestionably derived in by far the greater proportion of instances should only make us more desirous, more anxiously solicitous, to extend the same benefit to all. Influenced by these considerations, we cannot but feel that a careful, candid, and unbiassed investigation is required into many points which have either never been sufficiently cleared up, or are, from some cause or other, again involved in uncertainty; and this not only for the sake of truth, but for the minor, though no less pressing object of satisfying the public mind. Among these points that of RE-VACCINATION is one of the most important, involving as it does the consideration of the influence of time in weakening the protective powers of the primary vaccination. We gladly avail ourselves, therefore, of the valuable materials afforded us by the treatises of Dr. Heim, in the larger and more recent of which many other questions also, of almost equal importance, are brought under notice.

The smaller of these works is simply what its title states, an account of the results of the revaccinations in the King of Wirtemberg's military force, during the years 1833, 1834, and 1835. The larger and more comprehensive treatise embraces the whole subject, and is divided into eight sections, whereof the first four are devoted to a condensed abstract of the cases of small-pox, whether confluent, distinct, or modified, occurring in each of the four circles or provinces into which the kingdom of Wirtemberg is partitioned. These circles, or departments, are those of the Neckar, the Schwarzwald or Black Forest, the Jagst, and the Danube; and are again subdivided into what are termed Superior Bailiwicks, to each of which a medical officer is appointed. It is from the official reports furnished by the medical officers of these districts that the materials of Dr. Heim's work are chiefly derived.

The fifth section gives a nosographical retrospect, first, of the true small-pox in its various stages, with the irregularities to which these are liable; its sequelæ; the diversities of character in the accompanying fever; the variations in the appearance of the eruption; its complications with the varioloid disease and with cow-pox, &c.: and, secondly, of the varioloid disease, considered, in like manner, in the several stages of its progress; the variations in the character and form of the eruption, and in the nature of the accompanying fever; its progress in persons who have neither been vaccinated nor had previous small-pox; its complications with scarlet fever and measles, with true small-pox, and with cow-pox, &c. The sixth section is occupied with an account of the geographical diffusion of the small-pox contagion, and concludes the first part of the work. The second part, to which we propose chiefly to direct our attention, comprehends the seventh and eighth sections, respectively devoted to an account of the vaccinations and revaccinations in the five years from July 1831, to June 1836, inclusive.

Dr. Heim, following the official reports from which his materials are derived, considers, first, the number of the vaccinated (geimpften*), with or without success, within the period specified; secondly, the number of children, three years old and upwards, not yet vaccinated according to the regulations; thirdly, the number of authorized vaccinators; fourthly, the appearance of the pock among the cows; fifthly, the observations made upon the development of the protecting pocks, and upon the effects of their complication with other diseases; sixthly, the abuses observed in the public vaccinations, and in the mode of keeping the vaccination-books; seventhly, proposals for a more simple and less costly method of conducting the vaccinations.

1. There would seem to have been considerable difficulty in ascertaining the number of the vaccinated, with or without success, from the official reports, partly in consequence of deficient information in some of these reports, partly from the imperfect manner of keeping the vaccination-books. To obviate these defects, Dr. Heim has recourse to calculations, founded chiefly upon an examination of the rates of mortality in the first years of life, observing the numbers recorded among the deaths as having been vaccinated, and taking into account also the number of those who were attacked with small-pox previous to vaccination. These calculations, with some others connected therewith, are derived from such of the reports (forty-five out of sixty-four) as afford the necessary particulars, and are reduced to a tabular form, of which the general results are, that, in Wirtemberg, of 100 dying within the first year of life, 9·38 have been vaccinated; of 100 living born, 35·93 die within the year, and 52·36 reach their fourteenth year; of 100 deaths, 42·76 are children in the first year of life; and of 100 births, 3·98 are stillborn. The proportion of the births to the population is as 1 to 23·12, and that of the births to the deaths 1·23 to 1. From some of these data Dr. Heim draws the inference that the greatest possible extension had been given to the vaccine process although we cannot here follow him through all the steps of his calculation. What is of far more importance to our present object is the startling fact that, notwithstanding this greatest possible extension of the protecting influence of vaccination, the rate of infant mortality, as deduced from the five years to which the observations refer, is actually higher in Wirtemberg than in any other country, Holland excepted. Süssmilch asserts that, in populous cities, of 100 deaths, 30 are usually in the first year of life, and that the mean number is 26; the general average deduced from the extensive enquiries of Professor Rau,† is 24 per cent.; whereas, in Wirtemberg, of 100 deaths, 42·76 were in the first year. Dr. Heim, after calling attention to the fact that 36 out of every hundred born die before the termination of the year, makes no attempt at an explanation, but observes, that it would be an object of the highest interest to ascertain by comparison how far the remaining acute contagious exanthemata, and their sequelæ, may have taken the place of the small-pox in the work of devastation; a suggestion already made, under similar circumstances,

* The word strictly means the *inoculated,* whether with small-pox, cow-pox, or other matter, but is used throughout in the more limited sense of *vaccinated,* in which sense we shall also occasionally employ it; although we shall, for the most part, adhere to the more common practice, now arbitrarily but very generally adopted, of designating the giving of cow-pox *vaccination,* and of small-pox *inoculation.*

† Ueber die unnatürliche Sterblichkeit der Kinder. Bern, 1836.

by Dr. Watt, of Glasgow, though, as Dr. Baron asserts, upon insufficient and erroneous grounds.

Another point requiring notice, alluded to under this head, is that, among 208,322 children vaccinated within the five years, no less than seventy deaths occurred during the progress of the vaccine process; a proportion which is, notwithstanding, below that of Baden, where, of 98,198 children vaccinated in the four years from 1817 to 1820 inclusive, 79 died; so that, of 306,520 vaccinations, 149 proved fatal in some part of their course. This gives a proportion of one in 2057: small, it is true, in itself, but, when taken in the aggregate through an extensive population, giving rise to an actual mortality by no means to be disregarded. A portion of this mortality, however, as Dr. Heim clearly shows, must be considered as arising from accidental complications, occurring during the progress of the vaccine vesicle; and he takes considerable pains to prove that the rate of mortality is not greater than what is usually observed among children in the earliest age of life, during a like period to that over which the vaccine process extends. The cases which are more intimately connected with the vaccination he attributes to the puncture, considered either as a simple or poisoned wound, the danger of which is necessarily greater at an age when the process of dentition, the active nutrition and growth of many other organs and systems, and the excitability of the vascular and nervous systems, must exercise so much influence. This apparently fanciful notion of our author must be kept in view by our readers. We shall find it influencing many of his theoretical views of the nature and efficiency of vaccine protection, and leading to one most important practical suggestion.

2. The number of unprotected children, exceeding the age of three years, acknowledged by the official document, at the close of the government year 1835-36, was 271, chiefly contumacious, or belonging to families having no settled residence.

3. The number of gentlemen practising vaccination in Wirtemberg, in 1836, was 905, of whom 104 were physicians and 801 surgeons. To each individual vaccinator there fell, on the average, during the five years, 231 vaccinations, giving forty-six as the annual average number. On the first introduction of the practice of vaccination, it was thought, in Wirtemberg as elsewhere, that the operation, in whatever way performed, afforded an absolute protection against the destructive ravages of small-pox; whilst, at the same time, from its esteemed simplicity, the performance of it was intrusted not only to physicians, but also to midwives, bath-keepers, schoolmasters, and ministers of religion. In many instances even the fathers of families, setting aside the claims of experience and skill, conceived themselves competent to vaccinate their own children. These irregular proceedings on the part of ignorant and unprofessional persons were, however, soon put a stop to, and, by an explicit regulation, the practice of vaccine inoculation was committed to the *physicians*, in whose hands it remains, in many states, to this day; but in others, and amongst these Wirtemberg, has been, for the most part, transmitted to the surgeons.

With these state regulations we have in this country little concern, as, to all appearance, it will be long ere the legislature will deem the health and lives of the community fitting objects for their attention and care.

That the indiscriminate vaccination which has been practised in this country by ignorant or unqualified persons, with little or no regard to the condition of the subject, to the selection of the vaccine lymph, or to the progress and character of the vesicles formed, is to be regarded as one of the main causes of the frequent failure of the vaccine process, we are fully convinced; but, with the indisposition at present manifested by the government to intromit with all questions of medical police, we do not see how adequate protection can be afforded to the public. It is of little avail that persons eminently qualified for this and other services are at command, if ignorant assumption is permitted, on the one hand, to impose on those who are unable to judge for themselves; while, on the other, a miserable system of unjust economy is upheld by authority, by which real efficiency is sacrificed to any consideration involving a calculation of pounds, shillings, and pence.

4. The rarity of the vaccine disease amongst the cows, not only in this country but in France, and in many other parts of the continent, is well known to our readers. They will scarcely be prepared, therefore, for the statement made by Dr. Heim of its frequency in Wirtemberg. In the five years over which his report extends, no less than 274 cows affected with the disease, or at least with pustular or vesicular eruptions resembling it, were submitted to the inspection of the official authorities. Of this number, 188 were considered to have the true pustule, the others being rejected as spurious; and lymph was taken, for the purposes of inoculation, from forty of those pronounced to have the genuine disease. The distribution of this number of cows affected with the disease in the several departments is as follows:

| | Genuine. | Spurious. | Employed successfully in the inoculation. |
|---|---|---|---|
| Neckarkreis . . . | 56 | 21 | 18 |
| Schwarzwaldkreis . . | 72 | 41 | 8 |
| Jagstkreis . . . | 33 | 12 | 6 |
| Donaukreis . . . | 27 | 12 | 8 |
| | 188 | 86 | 40 |

It is worthy of remark, as the author observes, that in the Donaukreis, or circle of the Danube, an agricultural district, in which not only is the stock of cattle the largest, but also other circumstances favorable to the progress of the cow-pox are found to occur, the number of the affected cows was the smallest; and that, on the contrary, in the vineyard country of the Neckar, the number was so considerable.

The remark made by Reckleben, Thär, and others, that only cows affording fresh milk could become affected with the genuine pock, is not borne out by the observation of Dr. Heim; but the most important part of this subsection is the description given of the varieties of the spurious disease. Apart from the symptomatic pocky eruptions associated with the *Milzbrand* (malignant pustule) and other epidemic or infectious diseases, there are no less than eight peculiar forms of cow-pox (the spurious cow-pocks of Jenner) described by Dr. Heim, of which five are communicable to man. The three varieties which do not produce any appreciable effects upon the human system are—1, Viborg's wart-pock: this arises spontaneously in the alpine districts, and consists of red pustules, which contain a yellow humour (Feuchtigkeit), and run into wart-like indurations, often of long continuance. 2. The whitish tetter-like eruption of Thär, of the size of a pea, and surrounded with a small areola, the papulæ gradually succeeding each other, and developing a spurious cow-pock, which transforms itself into a deep-red ulcer with a blackish crust. 3. The white bladders known by the name of wind-pock, which contain a viscid fluid, crack easily, and form ulcers. The five varieties which are communicable to man, and readily give rise to local symptoms, are—1. The amber-pock of Nissen, with yellowish, almost transparent pustules, of the size of a kidney-bean, which exhale a cadaverous odour, readily burst, and run into corroding ulcers: the animals become feverish, refuse their food, and infect the hand which touches them with ill-conditioned ulcers. 2. Attended with milder symptoms than the preceding, but yet generating deep corroding ulcers in man, is the black-pock of Nissen, which is of a blackish appearance, and surrounded with a small red areola. 3. The blueish pocks (Var. *cærulea*, Niss.) occur most abundantly, are of the size of a pea, and surrounded by a small margin, and are accompanied with only a moderate degree of fever: they produce, however, only a small pustule upon the milker. 4. The white pock (Var. *alba*), with large vesicles filled with a yellowish ichor, induces swelling and inflammation of the hands of the milkers, with secondary ulcers. 5. The red pocks (Var. *rubra*) are of the size of a pea, and appear as red, hard knots, which pass into vesicles: these vesicles spontaneously burst, become indurated, or produce kindly ulcers, readily giving rise to local symptoms in man.

It is obvious that the occurrence of these and other modifications of eruptive disease analogous to the true vaccine, materially complicates the question of vaccine inoculation, at the same time that it demands for the safe and successful practice of the primary inoculation from the cow, a degree of experience which few, if any, have hitherto had the opportunity of acquiring in this country. At this time, too, more especially, when a spirit of enquiry is evidently at work, and when the genuine disease amongst the cows is stated to have recently made its appearance,* it becomes of the first importance that those who are anxious to introduce fresh sources of lymph should be fully aware of the difficulties which may in this way obstruct their path, and render their endeavours, to say the least, fruitless, if not positively injurious. But not only have they the spurious forms of cow-pox to contend with, but numerous modifications and anomalies in the genuine disease as it exists in the cow, affecting its transmission from the cow to the human subject. Some of these appear in the Wirtemberg reports.

A child was inoculated from a cow, from which not only four other cows in the same stall had been infected with the disease, but also an attendant had received the infection,—a pustule being produced in the middle finger of the right hand. For five days the punctures remained dry, and on the eighth day the child was vaccinated from the arm of another. Now *all* the punctures took, and as many as eight pustules were formed. A cow, on the 6th of December, was hot and had little appetite; soon after, small pustules with red areolæ formed upon the udder, but which on the 15th of December (the ninth day) had already become

* See page 591 of our last Volume.

encrusted, and no more clear lymph could be found, except in one which appeared to have arisen subsequently. This pustule was of a pale blue or lead colour, with an inflamed margin; it was seated on one of the nipples, but was smaller than the pock in children, not pointed, but round and flat, and contained a perfectly transparent and clear lymph, and no puriform matter. On the 16th of December, a child, seven months old, was inoculated from this abortive pock, and six perfect pustules formed upon the arms.

5. The observations upon the development of the cow-pox, and its complication with other diseases, are of great interest and importance, and require a more extended consideration than has been given to the preceding points of this investigation. Dr. Heim first notices under this head the reinoculation of those who have already been vaccinated without effect. The total number of children belonging to this class, according to Table ix., amounted to 2098 out of 208,322, or about one per cent. Of this number, the first Revaccination (or second vaccination) was unsuccessful in 68; the second Revaccination (third vaccination), in 77; the third Revaccination (fourth vaccination), in 4; and the fourth Revaccination (fifth vaccination), in 1; the intervals between the vaccinations being usually twelve months, though frequently not more than a few weeks. Taking into consideration every impediment to the reception of the infection, whether arising from the lymph itself, the time of year, or other cause, Dr. Heim thinks that not only the great number of primary failures, but, yet more, the repeated want of effect of the Vaccine, under the most varying external circumstances, would seem to show that in the first year of life an immunity from the cow-pox contagion may not unfrequently exist; that the susceptibility for the same may be first formed in the infantile organism, in which so many processes of development are at work, during or after the first period of dentition; and that it arises not always at once, but slowly and by degrees. Hence, he infers, comes the varioloid disease in children not yet vaccinated, or the appearance of a modified, or of only one genuine vesicle at the first vaccination of children, in whom, after a short time, a second vaccination, acting upon a portion of the susceptibility which had been kept back, may succeed, or again produce a modified effect: and hence the possibility that even regularly vaccinated children may, after a short interval, become affected with small-pox. The Vaccine has accomplished for them what it could; but it either acted upon a small proportional quantity of the already too abundant susceptibility to small-pox infection (vorräthige Pockenanlage), and was not in the condition to extinguish the whole amount; or it was applied during the formation of this susceptibility (Anlage), exhausted that already collected, but could not prevent the after-development of the unextinguished remnant. This is mere hypothesis: but this alternative affords an explanation, Dr. H. thinks, of all the irregularities hitherto belonging to the progress of cow-pox, and establishes the practical rule *that a small number of punctures, and the too-early vaccination of children, cannot be considered proper in all cases.*

From the foregoing observations it will be seen that the formation of a single vaccine vesicle, more especially in the first year of life, is not considered by Dr. Heim to afford sufficient protection; and several instances are related of the successful revaccination of those in whom one vesicle only had come to perfection. A child who, two years before, had been vaccinated, with the effect of producing only one, but a perfectly formed pock, was revaccinated by Dr. Klett upon these grounds. Two pustules, very perfect both in shape and progress, were generated, but were accompanied with only slight feverishness. Another child had been vaccinated with dry matter, with the formation of one vesicle only: reinoculation of the child from this same pustule furnished, in seven days more, two regular protecting pocks. A third child, in the district of Schorndorf, vaccinated from dry matter, had only one good vesicle, from which two punctures were practised upon the other arm. On the seventh day, two perfect pustules came to maturity. The following fact is curious, though not strictly of the same nature as the preceding. Dr. Uhl, in the early part of the year 1836, vaccinated fourteen children with matter from a child who had been brought out in rainy weather, and had been chilled in consequence. In four of these children the cow-pox regularly took effect, with the exception that the vesicles were somewhat too small, and the surrounding areolæ not of their true red colour. In the other ten only modified vesicles made their appearance, for the most part of the size of a pin's head, without either central depression or red areola. From these imperfect vesicles Dr. Uhl revaccinated the ten children, each with its own matter, upon the originally inoculated places; the pocks themselves being so small as scarcely to afford the requisite quantity of lymph for the inoculation; and a perfectly regular cow-pox vesicle appeared upon each. Altogether thirty-two individuals, each with one good vaccine pustule, underwent reinoculation; of whom the third part seemed to be not protected.

The subject of modified cow-pox,—that is, of cow-pox in which the insertion of vaccine matter is, from some cause or other, attended with only partial effects, the vesicles not proceeding in their due course, and never attaining to full perfection,—is one upon which much valuable information is given, affording support, as Dr. Heim thinks, to the views already alluded to respecting the progressive development of the vaccine and variolous susceptibility. A child, a year and a half old, was vaccinated, the first time with dry matter, the second time from arm to arm, but upon each occasion with imperfect success; finally, a third trial developed a good cow-pock, but the surrounding areola was not perfected until the twelfth day. Reasoning upon this and other analogous cases, Dr. Heim infers that the capability of receiving the pock is, in not a few cases, developed at a period *subsequent to the birth of the child;* and that vaccination, performed in the first months, and even first years of life, is often of little avail. The susceptibility, he conceives, when first awakened, gives rise to a modified form; and, as it becomes stronger, one or more regular and fully formed vesicles may be developed. Dr. Hartmann, at the vaccination of the year 1835, vaccinated a child a year and a half old: seven pocks were developed, of which five were genuine and two modified in their progress. In a child, thirteen months old, of six pustules, five were genuine and one imperfect. Again, in a child of one year, three perfect and two imperfect vesicles were formed. Other cases are reported, and adduced by Dr. Heim as favouring his views. We are, however, desirous, upon the present occasion, of laying facts before our readers rather than of discussing opinions; and must therefore be content with giving the following short extract as the conclusion at which the author arrives:*

"The experience of our inoculators affords favorable, and indeed unequivocal, testimony in support of my view, that the course of modified cow-pox bears the same relation to the true vaccine that the varioloid exanthem does to variola; and that it only expresses the approaching (annähernde) receptivity for the variol ous or vaccine virus (contagium); that, in fact, in the modified result of inoculation, nothing can be more incorrect than to place the varioloid exanthem in opposition to variola. As the latter (varioloid disease) is nothing but variola mitigated by reason of a diminished disposition to the reception of variolous disease, so the former (modified cow-pox) is merely the vaccine modified or mitigated by reason of a lessened or not sufficiently developed disposition for the reception of cow-pox; and they both possess only a temporary protective power, in proportion as they absorb the store of corresponding constitutional disposition." (p. 506.)

The following instance of the modified progress of the cow-pox is important, as showing the influence exercised upon the vaccine by peculiar states of the constitution. In a child who was affected with inflammatory symptoms of the chest, the vesicle was irregular in its progress, and on the eighth day rapidly dried up: when restored to health, the child was reinoculated, and with the best effect.

The usual practice of the inoculators is, it is stated, to place no reliance upon a modified success; very many of the reports making no distinction between complete and partial failures, and recognizing only the appearance of genuine vesicles as evidence of the protecting influence being in any degree exercised.

Passing over some instances of modified cow-pox induced by the vaccination of those who had previously suffered from small-pox and chicken-pox (Wasserpocken), we proceed to notice the retardation which is occasionally observed to take place in the progress of the cow-pox. This retardation of the regular progress of the cow-pox was not usually followed by any deviation from the normal appearance of the pustule; neither did the quantity of the vaccine matter employed seem to be concerned in producing it, since it was observed to occur as well when the inoculation was performed from arm to arm, as when it was effected through the medium of dried lymph; and indeed no less when the original cow-pox lymph itself was employed. Among the external circumstances which are reputed to exert an influence, the cold weather of the early spring is especially pointed out. In the district of Welzheim, in the Jagstkreis, in all the children inoculated in the cold month of March, the vesicles were two days later in making their appearance. Dr. Heim, however, is not disposed to attach much weight to cold as a cause of delay, since the retardation was not observed to occur more in winter than at other seasons, and he considers that delay of advance is rather to be ascribed to the individual force of reaction in the children, than to any external circumstance. Not a few of these retarded cases he states to have occurred when the number of pocks was not more than one or two. For the most part, the retardation of the cow-pox process was only for one or two days, but cases were observed in which it reached

* In translating this passage, we have paid more regard to the general sense than to verbal arrangement. There is here either a great want of elegance, or rather perspicuity of style, on the part of the author, or else, which is highly probable, (see note at the end of the book,) some omission or mistake on the part of the printer.—Rev.

from four and five to eight, and even to eleven and twelve days. Two children were vaccinated with dried lymph: on the eighth day there was as yet no trace of a vesicle; one of the children was again vaccinated with fresh matter, eight punctures being made; and fourteen days after the first inoculation there were as many as thirteen pustules, eight from the second, and five from the first inoculation. The vaccination was not repeated on the other child, but after fourteen days one pustule was formed, which, on the sixteenth day, presented the appearance usually observed on the eighth day. This child was afterwards revaccinated, on account of there being only a single pustule, but without effect. A child, on the eighth day after inoculation, had merely an obscure trace of the operation; in eight more days, two perfect pustules were to be seen. Many other examples are reported, some of which, as well as some of those above mentioned, we cannot but think, are opposed rather than favorable to the views of a progressively developed susceptibility advocated by Dr. Heim.

Occasionally, though not so frequently as the retardation of the process, the vaccine was observed to be accelerated in its progress. The influence of weather and season is, for the most part, alleged also as the cause of this anomaly. In several of the inoculated, in the district of Gerabronn, the vaccine was already fully formed on the third or fourth day; the pustules being filled with clear lymph, and regularly encrusted by the following day. This deviation was ascribed to the continued heat, from the effects of which the children, though not ill, were observed to be weak and to look pale. In the districts of Schorndorf, Nürtingen, and Heilbronn, the cow-pox was accelerated by one day; the warm weather being assigned as the cause. It should be stated, however, that the reports are not uniform in this respect, and that some instances are brought forward both of the acceleration of the vaccine in cold weather and of its retardation in summer. Dr. Becher, of Stutgard, considers the lymph to be less active in the heat of summer than in the colder seasons of the year.

The influence of atmospheric temperature on the development of the vaccine pustules, is a subject of great practical importance. The effect of cold in retarding, and of heat in accelerating, their progress must, we think, be admitted to a certain extent. Some striking illustrations of the fact were published some years ago by Dr. Howison, in the London Med. Gazette, (vol. 8.) They occurred in his practice in Edinburgh. But neither what is seen in this country or in Germany can give any adequate idea of the extent of influence which atmospheric distemperature really exerts. We must, for this purpose, learn from Griva and others the effects of vaccinating in Italy during the presence of a sirocco; in Egypt, while the hot winds blow from the desert; we must note the difficulty which is experienced in perpetuating a pure stock of lymph at Sierra Leone; we must learn from the truly *National* Vaccine Establishment of St. Petersburgh their comparative success in winter and summer; we must mark the increasing difficulties of the vaccinists in Hindostan, as displayed in the Transactions of the Medical and Physical Society of Calcutta. To enter at large on this branch of the enquiry is not our business or design; but we suggest it to those who have the opportunities as one which may usefully occupy their thoughts and engage their attention.

A question of very great moment in the practice of cow-pox inoculation is the age at which it should be had recourse to. The natural desire felt by the advocates of the operation, that its protective influence should be afforded at an early period of life, may possibly, in many instances, have led to a premature introduction of the vaccine virus. That this is Dr. Heim's opinion will be readily anticipated from what has gone before; and we are bound to admit that several of the reported cases would seem to give a degree of countenance to it. In children from three to four and even six months old, the pustules are stated to have been not only not so perfect as in those vaccinated at a later period, but the marks left by them are stated to become less distinct, and even to fade entirely away. While it is recommended, however, that no child should be vaccinated *within the first twelvemonth*, it is especially urged that none should be allowed to remain unvaccinated who have attained the age of three years. We have no reason to believe that this suggestion of our author has ever been acted on, nor can we easily be persuaded that there exist any good grounds for its adoption. Four months was the age at which variolous inoculation was most successfully practised, and we are convinced that the same period is equally fitted for the due development of the vaccine.

Considerable objection seems to be entertained on the part of some of the parents to permitting vaccine matter to be taken from the vesicles formed on the arms of their children. They affirm that it puts the children to considerable pain, and that it brings injurious consequences; and from the practice which appears to have been occasionally followed, of completely exhausting the pustules, it would seem that sometimes they have very sufficient grounds for their repugnance. Dr. Heim states that cases are known to him in which, after a copious abstraction of matter, an enormous erysipelatous swelling of the upper extremities, accompanied with irritative fever, attacked the children; and that in one of these cases, that of a child a year and a quarter old, death from convulsions ensued three weeks after this proceeding. Similar cases, he adds, have been frequently observed, and suggests that a restriction should be placed upon the arbitrary breaking up (Unterwühlens) of the pustules. One at least should be preserved intact, and the others should never be so rifled of their contents as to give rise to unnecessary suffering, or to the slightest risk of inducing inflammatory reaction.

We are perfectly in accordance with our author on this part of the surgery of vaccination. The local irritation which such a thorough exhaustion of the vesicles presupposes, is much to be deprecated. While we readily acknowledge this, we cannot, at the same time, omit to state, in justice to the practice of the late Dr. Walker, that his views were altogether different; that he never acknowledged this doctrine; and that his vaccinations have stood the test of *time* (the best of all tests) fully as well as those of Jenner.

Great stress is laid by the Wirtemberg physicians on the *number* of punctures, as it is conceived by many that, unless some evident constitutional effect is produced, little reliance is to be placed upon the protective powers of the mere local affection. Hence, as we have already had occasion to show, when only a single vesicle comes to perfection, it is a very general practice to revaccinate. The number of incisions, however, varies considerably in different places: some of the inoculators contenting themselves with from two to four upon each arm, while others endeavour to produce as many as fifteen, twenty, and even thirty pustules. This extreme pustulation, however, excites very severe constitutional disturbance, and produces a morbid state of no trifling import. Since the year 1831, it has been the practice of the inoculating physicians in the district of Ulm, to increase the number of pustules; and they have observed that, when a considerable number of pocks are developed, a primary fever appears as early as from the third to the fifth day, which, with a smaller number of vaccine pustules, is scarcely, if at all, perceptible: whence, says Dr. Heim, it may be concluded, with Dr. Gramm, that the increase in the number of pustules renders the vaccine disease more severe, and that the susceptibility to the small-pox contagion is, in consequence, thrown off for a longer time. Dr. Camerer endeavoured to increase the number of pustules to at least sixteen, and for the most part with success; he observed also a stronger primary fever to accompany the increase. This practice seems to have been pushed to its greatest extent by Dr. Wanner, of Oehringen, who for many years had been in the habit of making an increased number of punctures, under the idea that it was the most certain means of affording protection from subsequent attacks of small-pox. Of 505 children inoculated by him during the government year 1834-35, three fourths had as many as from fifteen to twenty, and some even upwards of thirty pustules each, and with the best success; to which also the goodness of the cow-pox matter probably contributed its share. From this increase in the number of the pustules, the cow-pox fever was much more severe, and the parents complained that, since this method had been adopted, the fretfulness and restlessness of the children were much greater than before; that the heat was very great in the night; and that such feverish excitement became apparent between the third and fourth days, an occurrence which they had never remarked in the earlier inoculations, when the pustules were fewer in number.

The number of punctures, however, would seem to have been about three on each arm in the general practice of the inoculators, and from these from four to five pustules were developed, on the average. The opinions expressed by Dr. Heim himself on this practice of greatly increasing the number of punctures are judicious, and highly deserving of the attentive consideration of its advocates. "I have," he says, "pointed out that twelve punctures are sufficient for revaccination, and an equal number also may be thought sufficient for the vaccination of the young. Though many inoculators may have recourse to a greater number of punctures in children, without local or general detriment, and have advised the general adoption of this practice, several facts have, nevertheless, been recorded, from which it would seem that too many punctures have been followed by severe local inflammation and gangrene, dangerous irritative fever, convulsions, and (according to Vogel, Lyner, and others,) even death itself. It is probable, therefore, that the number of from twelve to twenty punctures, required by Eichhorn and Gregory, may mark the minimum and maximum, and that the doubling the usual number of incisions, as amongst us, is more than sufficient." (p. 519.)

We have thus given, at some length, the opinions of our German brethren on two great questions connected with vaccine pathology,—the increase of protective power by increase of vesicles, and the necessity of developing fever so as to ensure the full measure of protection. The latter doctrine has never been generally adopted in this country, but it has received the able support of Staff-surgeon Murray, in some papers published by him in the London Medical Gazette. His experience was obtained at the Cape of Good Hope. With regard to the first, it appears to us that, provided the lymph employed be of an energetic quality, two, or at furthest three, insertions are amply sufficient for the security of the individual; though more may be useful with reference to the convenience of public supply. The numerous punctures alluded to as having been formerly made by Dr. Gregory, at the Small-pox Hospital, (amounting often to twenty or thirty,) were adopted at a time when the virus was very weak and of small intensity. The practice has been wholly discontinued since the more energetic virus now employed came first into use. A certain degree of sympathetic fever (though not, as we conceive, essential to vaccine protection,) is yet desirable, as giving undisputed evidence of constitutional affection. We know not what is the nature of the salutary change actually effected in the system by the insertion of the vaccine; but it is more reasonable to think that a high degree of fever should disturb, rather than that it should promote that desirable modification of temperament.

In accordance with the preceding opinion as to the importance of a certain degree of constitutional action being induced, is the ascription of superior efficacy, by some of the Wirtemberg vaccinators, to lymph procured direct from the cow. It is certain that, in cases of inoculation with primary vaccine lymph, there is a much more severe local affection and a greater reaction manifested than where the lymph has repeatedly passed through the human subject; an observation which has been made not only by the foreign authorities, but also by many in this country. Some of these reports also describe the pustules from primary lymph as being finer and of a peculiarly genuine appearance, and attended with swelling of the axillary glands, and in a few cases with severe secondary fever, vomiting, and convulsions. There would seem, however, to be more difficulty in inducing the disease by the insertion of original lymph than by vaccination with that which has already been assimilated, as it were, to the human constitution; a considerable proportion (perhaps three fourths) of the inoculations made with the lymph direct from the cow altogether failing in effect.

Some curious results are given of the simultaneous insertion of lymph direct from the cow on the one arm, and of lymph from the human subject on the other arm, by the District-surgeon Fehleisen, in the year 1832. The experiment was repeated in the spring of the year 1836, in a similar manner, and with analogous effects, by M. Bousquet, at the instance of the French Academy. For the details of the original experiment we must refer to Dr. Heim's work: the results obtained by M. Bousquet, which are the more important as having been witnessed by several of the faculty of Paris, are as follows: About fourteen days after the breaking out of the genuine pock on the udder of a cow at Passy, near Paris, M. Bousquet inoculated several children, some with this lymph alone, others (for the sake of comparison) with the new lymph on one arm and with the ordinary vaccine on the other. The woman who milked the cow, and who had vaccine pustules on the fingers and on the lips, together with three of these children who were ailing, weakly, and miserable, were shown to the Academy and the members of the Vaccine Commission, on the 30th of March. Pustules, but equally perfect on both arms, had formed upon all the punctures. Several children were inoculated from these, on the left arm with the new cow-pox matter, on the right with the old lymph. On the 5th of April, three of these children were inspected at the Academy, by a great number of physicians; and upon this occasion with a different result. A pustule had formed upon each puncture: those from the new cow-pox were more perfectly developed, larger, flatter, clearer, and contained a more limpid and more abundant fluid ; while those from the old lymph were smaller, more elevated, of a paler and yellowish colour, and contained a deeper-coloured, clammy fluid. Considerable difference of opinion as to the cause of this diversity seems to have arisen ; some of the members of the Academy concluding that the original lymph had degenerated, and that its protective powers had become either weaker or modified: others were not disposed to attach much importance to differences occurring in the size and elevation of the pustules, their development being influenced by manifold circumstances; such as the varying states of the atmosphere, idiosyncrasy, the period at which the lymph is taken, &c. The two series of experiments with the new lymph also, as M. Bousquet himself allowed, afforded different results; the children who were inoculated with both kinds of lymph showing finer and larger pustules from the new than from the old, while the children who were inoculated with the new lymph only presented pustules altogether of the usual appearance.

"The experience of all ages," observes Dr. Heim, in conclusion, "teaches that contagion, in its progress from body to body, becomes, after a time, milder and less fatal; as in the plague, the yellow fever, syphilis, leprosy, &c.; and certainly, notwithstanding the opposite views entertained by the English and French physicians, the vaccine also cannot be altogether excluded from this analogy; while, on the other hand, the Germans again may have been in error, by setting forth the deterioration of the unrenewed matter in too forcible a point of view. Be this however as it may, this much is very certain, that the eventual operations of both kinds of matter is the same: the lymph taken immediately from the cow, even in the inoculations performed under the eye of Jenner himself, could not prevent the renewed susceptibility for the small-pox contagion, and protected neither longer, nor indeed more nor less, than the assimilated (humanisirten) cow-pox. A like fate will probably in time attend all later inoculations from the cow, if care be not taken to give renewed protection by revaccination. The places most abundant in original lymph, Switzerland and Holstein, exceed other countries in the frequency and extent of small-pox and varioloid epidemics. It will, therefore, be always advisable that too much value and reliance be not placed upon the renewal of the vaccine matter; and while this, when opportunity occurs, should not be neglected, yet, in regard to the measures relative to the protection against the return of the small-pox, it may be left wholly out of account." (p. 524.)

The length to which this analysis has already extended, and the important matter which still remains before us, preclude us from entering into this and other questions of the highest interest alluded to in the course of the preceding observations; but it must not be allowed to pass entirely without notice, that the foregoing conclusions of the author are scarcely consistent with the views which he has elsewhere advocated, or with the facts repeatedly brought forward. The most striking defect, as it appears to us, in recent vaccinations, more especially in those practised in this country, has been a too great reliance upon the character of the local affection *alone*, without any regard being had to the production of an effect upon the general system. This opinion, setting aside the speculative notions as to the exhaustion of variolous or vaccine sensibility, and the subsequent renewal of the same, agrees in the main with the views of Dr. Heim and many of the Wirtemberg physicians. Now if, as would seem to be the fact, more severe constitutional symptoms are developed in connexion with the vaccine as induced by lymph directly from the cow, we can scarcely subscribe to the opinion that, in regard to the protective measures against the return of small-pox, primary or direct vaccination can be left wholly out of account. On the contrary, if an equal degree of constitutional affection can be induced by from two to four or six primary vesicles as by from twelve to twenty or more punctures with assimilated lymph, an evident advantage would be gained in the diminution of the amount of suffering and of the purely local inflammatory symptoms, to which, after all, much, if not the whole, of the constitutional disturbance in these over-scarified cases may be owing.

The effects of the complication of the vaccine with other diseases are entered into at considerable length, and are well worthy of attentive study. Brief notices of the more important instances are given for each year of the period to which the observations extend, and afterwards a general summary of the results, classed under the respective heads of the complications with heterogeneous fevers, with acute exanthemata, and with chronic eruptions. We regret to be compelled to pass over this part of Dr. Heim's work in a very cursory manner. Two or three points, however, to which considerable importance at one period or another has been attached, call for remark. One of these points, important on account of its universal occurrence, is dentition. Dr. Heim thinks that this process is not usually delayed by the vaccine disease: on the contrary, in many cases it was observed to be accelerated by the fever attending the development of the cow-pox, and he states that within the five years no less than six children died during the course of the vaccine, from the more energetic progress of the dentition. The vaccine process itself does not appear to have been interfered with by the simultaneous development of the teeth.

We confess we are rather surprised at this statement; for we have always been of opinion that dentition was one of the things that most materially interfered with the process of vaccination. The pathological principle involved in this consideration we believe to be this, and it is very important and of wide application :—Extraneous fever, however excited, prevents the growth and checks the normal progress of the vesicle. If the variolous germ be received into the system quietly, it may advance, *pari passu*, with vaccination, and neither interfere with nor modify the effects of the other. But the moment fever is excited, that moment the vaccine vesicle begins to droop. So with regard to dentition: if it go on calmly, the vaccination is not influenced; if fever be awakened, the vesicles suffer.

Among the exanthematous eruptions, passing over the complications with measles, scarlet fever, erysipelas, &c. (the first of which especially would seem to be rendered milder by concurrence with the vaccine,) those which are of a pustular or vesicular character require a brief notice. Varicella (water-pox, stone-pox, wind-pox,) is said to be often epidemic at the period of the public vaccinations, and is consequently frequently observed with the vaccine; sometimes exerting no influence upon its progress, sometimes either accelerating or delaying the formation of the vesicles. Children scarcely recovered from varicella were vaccinated with good effect; and, again, in other cases, the water-pox (the purely vesicular disease, or chicken-pox of Dr. Alison?) followed the vaccine, and both appeared to tread the one upon the other, entirely without correlation, unless perhaps a power of awakening the chicken-pox should be conceded to the insertion of vaccine matter. An argument is subsequently attempted to be founded upon this circumstance, against the identity of the water-pox and the variolous contagions; but, for a fuller examination of this matter, we must refer our readers to Dr. Thomson's "Account of the Varioloid Epidemic in Edinburgh," (with which work, by the way, Dr. Heim seems to be but imperfectly acquainted;) to Mr. Cross's "Sketch of the Varioloid Epidemic of Norwich," and the other works which profess to unravel that long-disputed question.

A peculiar secondary eruption, by many named the cow-pox eruption, is described as having been observed to accompany the vaccine process: it appeared under different forms, sometimes only partial, but occasionally spreading over the whole surface of the body, passing through various gradations, from a spotted measles-like rash to the papuliform, vesicular, or truly pustular character. It did not retard the vaccine process, nor disturb the general health, with the exception of occasionally giving rise to a marked increase of the fever. It usually occurred between the eighth and fourteenth days, and lasted for two or three days. In the greater number of cases, the form was that of the miliary kind, showing small, reddish, cuticular elevations, which soon gave place to a bran-like desquamation. That which appeared after the fourteenth day of the inoculation assumed the character of what Gölis terms the cow-pox itch, which consists of isolated vesicles often filled with a puriform fluid. These vesicles, after bursting, become covered with scurfs, or leave behind superficial ulcers, some of which often take a long time to heal. In a few cases the eruption assumed the appearance of chicken-pox, and in six cases a few vesicles, partially resembling the cow-pox (modified) or entirely similar to it, showed themselves in different parts of the body from the seventh to the ninth day after the inoculation. This description of the several forms of constitutional vaccine eruption appears to us to be elaborately and carefully drawn up, and it affords a favorable specimen of the talent for patient research which characterizes our German brethren.

Fifty-four cases of the complication of the vaccine with variola were observed; 28 of which occurred in those vaccinated for the first time, and 26 in revaccinated individuals. Of the first 28, or primary vaccinations, 24 were children (for the most part under the age of twelve months), 17 had variola vera (of whom 7 died), and 7 varioloid disease (of whom 1 died.) Of the second 26, or revaccinations, 3 only were variola vera (of which 1 died), and 23 were the varioloid disease (of which not one proved fatal.)

It is worthy of especial notice that from a child belonging to the first section, three quarters of a year old, who was seized with small-pox on the fourth day after the vaccination had come to maturity, several other persons were revaccinated, in seven of whom the vaccine succeeded, but without a trace of variolous contamination.

Of the chronic eruptive diseases, it is observed, that but seldom was the regular progress of the vaccine either limited or hastened by their presence, and yet more rarely was any modification of the cow-pox produced; neither as a general principle was there any retarding influence exercised upon the vaccine pustulation. The effect of the vaccine upon the chronic eruptive diseases seems however to have been beneficial, rendering them milder in most instances, and very often exercising a healing effect, so that in not a small number of cases the fall of the cow-pox crusts was accompanied with the healing of the long-standing and nearly-habitual skin disease. The itch, with which many children belonging to different districts were affected when vaccinated, for the most part did not retard the vaccine process. Herpes (Flechten), under which only a few children laboured when vaccinated, usually produced no alteration in the vaccine pustule; in one child affected with herpes, there appeared two modified pocks.

The conclusions at which Dr. Heim arrives upon the subject of complications are, that "from the united observations of the five years it appears to be established that inflammations, inflammatory fevers, dentition, influenza, &c. assist and hasten the progress of the vaccine; that, on the contrary, nervous fevers, epistaxis, and other hemorrhages, diarrhœa, dysentery, and cholera may weaken and retard the process; but that acute and chronic eruptions usually exercise no essentially modifying influence, while these last affections are themselves rendered milder in their course by the presence of the vaccine disease." (p. 555.)

To those who have read and studied the later writings of Jenner, these observations of Dr. Heim will necessarily appear very startling. Jenner taught that in preoccupation of the skin by any disease capable of affording *a humour*, whether thick or thin, serous, purulent, or glutinous, was to be found the *grand impediment* to efficient vaccination. It would lead us too far from our present object to enter into the merits of this theory, but it is clear from what has been just stated that our continental brethren join issue with Dr. Jenner on this point. And here we must express our unqualified disapprobation of the practice of inserting the vaccine virus for the purpose of affording a protection against small-pox, when any such disease exists on the surface of the body, or may be presumed to be lurking in the system. Indeed, on the contrary, we hold it to be an indisputable axiom, that unless circumstances imperatively call for immediate vaccination, the state of health of the recipient should be as perfect as possible; and we are inclined to attribute very many of the failures in protective power observed in this country and elsewhere, and some part of the mortality during the vaccine process occurring in the practice of our continental brethren, to a neglect of the requisite precautions on this head.

The remaining subdivisions of this section need not detain us, and we pass on therefore to the eighth section, which professes to give an account of the REVACCINATIONS in the kingdom of Wirtemberg during the stated years, 1831 to 1836 inclusive.

Dr. Heim, having given a hasty sketch of the progress of the vaccine theory, from the first announcement of the cow-pox as affording a perfect immunity against small-pox, (in which, however, as it appears to us, he scarcely does justice to Jenner, and indeed would rather seem either to have not sufficiently comprehended his views or to have misunderstood their general import,) proceeds to trace the gradual alteration in this respect which has, for the most part, taken place in the opinions of many even of the warmest supporters of the practice of vaccination. From this brief history, it appears that as early as the year 1829, the Wirtemberg government had recognized the principle of revaccination in cases presumed to be inadequately protected by the primary inoculation, at the same time that the appearance of the marks left by the first vaccination, was considered to afford the required evidence as to the fact of the constitution being effectively protected or otherwise. Cases, however, occurring in which, notwithstanding that the inoculative marks presented the required characters, small-pox, either in its genuine form, or modified, presented itself, it was subsequently recommended by the Royal Medical College, that in infected houses and their neighbourhood the revaccination should be had recourse to indiscriminately, without respect to the appearance of the cicatrix or to the age of the individual, a measure which was, for the most part, wherever practicable, adopted, and which was also carried into effect among the military.

Investigation into the subject of the vaccine cicatrix, considered as affording evidence of the protected or unprotected state of the constitution, has led the Wirtemberg physicians to conclude that little or no reliance is to be placed upon the sign in question. It was ascertained that the pustules of genuine cow-pox (analogous in this respect to small-pox) may leave an imperfect mark, or even no mark at all; and, on the other hand, that to depend upon the regular appearance of the mark as a sign of protection, was only to adopt a dangerous error and to lull the public into a state of false and injurious security. The circumstances attending the epidemic prevalence of small-pox, as well as the effects of the revaccination, prove that the cicatrix theory is untenable. Of 1055 cases of small-pox in which the marks were visible, 914 had good, and only 141 imperfect marks; 147 of these, notwithstanding that the vaccine marks were normal, were cases of genuine small-pox. Neither did the characters of the marks afford any criterion for the success of the revaccination process. In the district of Böblingen, of 2718 individuals of different ages who were revaccinated, 1322, or nearly one half, had regular cicatrices from the primary vaccination; yet of these last, in 65 per cent., the revaccination completely succeeded; in 26 per cent., the process was modified; and in 9 per cent. only did it fail altogether; 1134 of the whole number showed imperfect marks, and yet the revaccination failed in 18 per cent. of these, while it was modified in 28 per cent., and perfectly succeeded with the remainder. The results among the military, which are the more striking on account of the absolute reliance to be placed upon them, were, that of 14,384 revaccinations, 7845, or more than half, showed normal marks of the preceding vaccination, and yet of this number the success of the revaccination was complete in 31 per cent., modified in 29 per cent., and failed altogether in 40 per cent. In 46 per cent. of those with imperfect marks, the revaccination was notwithstanding without effect, while in 26 per cent. the success of the operation was modified, and in 28 per cent. complete.

It has already been observed that the genuine vaccine vesicle may occasionally leave no permanent trace of its existence, and accordingly it was noticed that persons presenting no mark of previous vaccination were not always to be considered as unprotected. The number of these cases is stated to be too great for it to be supposed that they had been previously affected with small-pox which had left no trace behind it, or for the circumstance to be attributed to idiosyncrasy, or to want of susceptibility for the vaccine. Of 127 cases of this description, (in which there was no trace of a cicatrix,) submitted to vaccination in the district of Böblingen, the result was successful in seventy-five, modified in forty, and in twelve the operation was without effect. Of the 14,384 revaccinated soldiers, 2030, nearly the seventh part, had no mark of previous inoculation, and of these 2030 individuals the vaccine succeeded in 34 per cent., was modified in 19 per cent., and altogether without effect in 47 per cent., or nearly one half of the number. A similar analysis, illustrated by a table, is given of a certain portion of the military in whom the number of the marks was ascertained, and with similar results; so that if we are to place any dependence upon the correctness of the statements given, and upon the care and skill of the inspecting physicians, the inference seems unavoidable, that the appearance of the cicatrix affords no indication whatever as to the effect of the previous vaccination in limiting the success of a second vaccination; and that it is of little value as indicating the amount of protection afforded against subsequent attacks of small-pox seems likewise clear, although this latter conclusion rests upon evidence, neither of the same amount, nor of the same character as the former. It should be observed, that a continued or renewed susceptibility for the vaccine, is by no means proved to be commensurate with, or correlative to, a continued or renewed susceptibility for small-pox; neither has it hitherto been established, at least to our satisfaction, notwithstanding the high regard we entertain for the opinions of many of those who maintain this view, that small-pox and the vaccine are so essentially and circumstantially identical, as to admit of this convenient method of reasoning in parallels. Without absolutely denying that it may be so, we should rather wait the result of further evidence before subscribing to the correctness of the opinion; and, in accordance with the wise practice of the Scottish Jury Courts, record, for the present at least, a verdict of *not proven*.

That considerable difficulty should occur in the endeavour to promote the practice of revaccination throughout the general mass of the population, will not be a matter of surprise. Various motives originating in supineness and indolence on the one hand, or in dislike and opposition on the other, unless the danger is imminent at the door, will always operate against every measure of utility which is not manifestly accompanied with present benefit. It is, therefore, no more than what might have been reasonably anticipated that, for the most part, with the exception of districts in which the variolous and varioloid forms of disease were prevalent and fatal, the progress in the revaccination of the civil part of the population has been partial. Yet, upon the whole, the result of the revaccinations in Wirtemberg during the five years has been in the highest degree successful. Of somewhat more than 44,000 who have undergone the operation, upwards of 20,000 were revaccinated with good effect, about 9000 with modified success, and the remaining 15,000 without effect. The following are the comparative results in the several departments, and amongst the military. Of each hundred revaccinations, there were

| | Good. | Modified. | Unsuccessful. |
|---|---|---|---|
| In the Neckarkreis . . . | 57 | 23 | 20 |
| In the Schwarzwaldkreis . . | 29 | 26 | 45 |
| In the Jagstkreis . . . . | 70 | 5 | 25 |
| In the Donaukreis . . . . | 27 | 35 | 38 |
| Throughout the Departments | 51 | 18 | 31 |
| Among the Military . . . | 34 | 25 | 41 |
| Of the total Revaccinated | 46 | 20 | 34 |

The difference observed in the four departments, in respect to each other and to the military, is thought to depend chiefly upon the different ages at which the revaccinations take place; thus, in the departments of the Danube and the Schwarzwald, in which the revaccinated were for the most part children, the proportion of cases in which the operation succeeded was smaller; in the army, where almost all those undergoing the operation are nearly of the same age, (twenty-one years,) there was a medium proportion of successful results; whilst in the Jagstkreis, where the persons who were revaccinated were generally more advanced in life, for example of the age of thirty years, the proportionate success was greater. It is scarcely necessary to observe that, according to the views of Dr. Heim, the more complete the success of the revaccination the greater had been the receptivity, not only of the vaccine but also of the variolous contagion, and the more completely had that susceptibility been, by the second operation, removed.

It has been already stated that the marks of the previous vaccination, although performed with complete success, were at the revaccination often found to be very imperfect, sometimes presenting nothing more than a scarcely visible clearer-coloured spot on the skin, and that even, in a few cases, not a trace could be detected of the success of the operation; yet the cicatrices left after complete success of the revaccination, appeared for the most part indented and strongly marked, although perhaps much smaller (not much exceeding the size of a pea,) than those left by the operation in childhood. Traces upon the skin were also usually observed from the modified success of the revaccination, though yet more imperfect than where the success was good, merely pale spots about the size of a lentil being visible, without sharpness of edge or depressed points. In the spring of the year 1836, fifteen recruits marked with spots of this description presented themselves at the reinoculation, stating that these marks were the remains of a former *revaccination*, in which the success had not been complete. In two of these, in whom the revaccination had been performed two years before, the progress in the year 1836 was again modified; of four who had been revaccinated three years previously, the success of the renewed operation was good in two and modified in two; in three who had been revaccinated five years before, the third vaccination succeeded in two and was modified in one; in one after seven years the renewed operation succeeded, and in all the remaining cases, of which two had been revaccinated eight years and two nine years previously, the progress of the third vaccination was modified. On the other hand, thirty-two individuals showed cicatrices, varying in number from one to six, which were of a better form, more clearly marked, and punctuated, although only of the size of a pea; from which it was inferred that the revaccination performed at their own homes had been attended with good success. Two of these had been revaccinated two months; six, one year; four, two years; seven, three years; and two, four years, previously: in not one of these did the renewed inoculation in the year 1836 succeed. In one who had been revaccinated five years before, the revaccination of 1836 had modified success; in another, as well as in one who had been revaccinated six years previously, the renewed operation was without effect; and in two after seven, two after eight, and four after nine years, the reinoculation was unsuccessful.

With respect to the duration of the protective power of revaccination, Dr. Heim thinks that the age usually selected for the performance of this second operation has left behind either the whole, or at least a large part of the morbid processes of the age of childhood; that the whole constitution has now acquired a greater degree of firmness, and is less liable to be affected by external circumstances than the mobile organism of infantile life; and, consequently, that there is no tangible reason why the revaccination should not extend its protection for at least an equal period of time with the primary vaccination, but under the following indispensable conditions: "1, that the success of the revaccination be complete, and in no respect less perfect than the good cow-pox of children; and 2, that the vaccine virus be in sufficient quantity to saturate (aufgedrungen) the system." For the fulfilment of this last condition, the average number of twelve punctures is thought sufficient, as has been before remarked, and for reasons which are stated more fully in the account of the revaccination of the military.

The following are the results of the revaccination observed in 152 individuals of different ages, in sixty-one of whom the operation succeeded, but in ninety was of no avail. 1. In the greater number the virus produced no effect, the abraded cutis healing in a few days. 2. In some the epidermis was raised in a few days into a small vesicle, which, however, sank again as rapidly and dried up without exciting any reaction, leaving no trace of its previous existence. 3. In others, the inoculation seemed to take effect, the pustules rose, became pitted, and filled with a transparent fluid, but quickly dried up as early as the seventh or eighth day, when usually the surrounding redness spreads, and the crusts fell off without leaving the characteristic marks; the constitutional reaction in these cases, with the exception of local symptoms in the axilla and down the arms, was unimportant. 4. Only in those individuals in whom the vaccine went through its regular progress, was any reaction affecting the general health observed.

In many corpulent persons, Dr. Bardili observed on the sixth or seventh day after the revaccination, transparent pustules, surrounded with a dusky blueish redness, in which the characteristic marks of the genuine protective pock were absent; in this form swelling of the arm and of the axillary glands, and marked fever always prevailed. These pustules commonly burst on the seventh or eighth day, leaving a painful, easily bleeding ulcer, a line in depth and varying in size, which in the course of from fourteen to twenty days became covered with a dry adherent crust. Other varieties were also observed which we cannot here particularize. Another report mentions that, in corpulent, strong men of dusky complexion, the inflammation ran high and the pocks were very full, becoming purulent as early as the eighth day. The inflammation of the skin was so extensive, that the individuals were unable to continue at their work, and were obliged to remain in bed. The same circumstances were also observed in the district of Oehringen; the fever was so severe that the patients were obliged to lie in bed; and the greater the interval between the first and second inoculations, the more perfect were the pocks, and the more severe the fever. It seems to have been very generally observed, that the shorter the interval between the two vaccinations, the less was the amount of the success which attended the second insertion of the lymph; while usually, and only with very few exceptions, was the revaccination entirely without effect when performed from five to six years after the first inoculation. It succeeded also, according to Dr. Rösler, in adults more certainly with lymph taken from adults, while with lymph taken from children it was frequently of no avail.

We have no great confidence in the correctness of this last observation, for it has been disproved again and again under our own inspection, until we have been tired of renewing the experiment; but the former alleged fact, touching the better development of the secondary vaccine vesicle in proportion to the distance of time that has elapsed from the first insertion of the virus, is, we believe, quite correct *in the main*, though liable, of course, to many modifications and exceptions. This has always appeared to us a fact of great importance, tending to establish the doctrine of a gradual impairment of vaccine protection.

Various degrees of modification in the revaccination process were observed in different places. In the district of Böblingen occurred several cases of this description:—1. The pocks formed sooner, or their progress was earlier completed, than in the genuine affection. 2. Although they put on the same form they did not attain the same size as the genuine pocks, which was particularly evident in the incrustation. 3. They filled after their appearance with a thin lymph, which remained as clear as water and limpid to the seventh or eighth day. 4. The areola was paler than in the genuine pocks and seldom round, and attained its greatest limit towards the eighth or ninth day, after which it usually disappeared rapidly. 5. The deeply seated induration accompanying this inflammatory circle was always less than in the true pock. 6. From the third to the seventh or eighth day the modified cow-pox was always accompanied with painful sensations in the axillary glands, attended by marked shiverings and occasional headach. In the district of Waiblingen the progress of the vaccinoid affection was observed to be quicker than the genuine cow-pox; usually there was itching in the wound a few hours after the revaccination, which about the third day became almost unbearable.

The pock was commonly fully formed about the fifth day, and contained a yellowish, viscid lymph; the pustules had scarcely perceptible cells, and not always the central depression; and the characteristic surrounding redness was wanting. On other occasions, the entire upper arm became highly inflamed, or red indurated streaks were observed; the axillary glands were painfully swollen; the period of suppuration was sooner completed; the crusts were deep seated, had no central pit, and were easily and earlier detached; and, lastly, the cicatrices were more superficial, not shining, and often uneven.

We have now to examine the effects of the vaccination upon those who had already suffered from small-pox. Of 297 persons marked with the small-pox who willingly submitted themselves to this operation, at the revaccination of those who had previously had cow-pox, the vaccine took effect regularly in ninety-five, was modified in seventy-six, and failed altogether in 126, giving a proportion of thirty-two in the hundred of the genuine form, twenty-six of the modified, and forty-two in the hundred of failures. A remarkable correspondence exists between these numbers and those afforded by the revaccination of the military, which however Dr. Heim fails to notice. The proportion of one hundred cases of each description is as follows:

| | With Success. | Modified. | Without Effect. |
|---|---|---|---|
| Vaccinated after Small-pox . . | 32 | 26 | 42 |
| Revaccinated Soldiers . . . . . | 34 | 25 | 41 |

Are we to assume, therefore, upon the principles advocated by Dr. Heim and the Wirtemberg physicians, with respect to the vaccinated, that of every hundred individuals who have gone through the natural small-pox, and that too in so severe a degree as to leave marks behind, no less than fifty-eight again become after a time liable to the disease? The author gives some statements, as to what has been hitherto observed in different countries with respect to this point, which are worthy of comparison with the preceding. La Condamine estimates the proportion of the secondary attacks of small-pox to the primary ones as 1 in 50,000; Heberden as 1 in 10,000; other English physicians as 1 in 8000; Eichorn again states the proportion as high as 1 in 250; and in the Copenhagen epidemic, according to Möhl, one of every six attacked with small-pox had before gone through the disease. This, however, by no means proves that one-sixth of those who had formerly had small-pox were a second time attacked; whereas upon the assumption, that those who are capable of being vaccinated with effect, either after small-pox or after the vaccine, are unprotected, more than one half would seem to become after a time susceptible, and nearly one third liable to the disease in its most genuine form. That we have not here forced an inference from the facts to which the author would not himself subscribe, is evident from his concluding remark. "Most certainly," says he, "might these individuals, vaccinated after small-pox, ninety-five with good, and seventy-six with modified success, have been attacked under an epidemic influence for the second time with one or other form of that disease."

Among the subjects subsequently examined in this section, is the value of revaccination matter, (that is, of matter which has been taken from genuine cow-pox pustules developed in a previously vaccinated individual,) for the purposes of further inoculation. Several objections have been urged against the employment of matter of this description; among others the doubt as to its genuineness, and the danger of conveying other poisons into the constitution with the vaccine virus taken from adults, for instance, those of syphilis, gonorrhœa, itch, &c. This last, as it appears to us, is an objection of no mean weight, but we cannot here enter into the subject further than to give a caution against the employment of the lymph derived from adults, and from those of weakly or diseased constitution generally. Dr. Heim, however, entertains no such fears, and states it as his opinion, that the vaccine virus is scarcely susceptible of mingling with others of similar nature. He says that inoculations have been practised with vaccine lymph from children known to be affected with itch, tinea capitis, and chicken-pox, or even when the vaccine has been complicated with genuine small-pox, (of which, indeed, an instance has been already alluded to,) without either of these diseases being transmitted to the inoculated individual, or the progress of the cow-pox itself interfered with. We must, however, state that the earlier vaccinations which took place in this country at the Small-pox Hospital of London, are decidedly opposed to this statement of Dr. Heim, and we trust that the vaccinations in this country will never be contaminated with any such, to say the least, doubtful practices.

That a considerable number of those who have been vaccinated in early infancy have subsequently become the subjects of small-pox, either in its genuine or modified form, cannot, after recent experience, be doubted; and that the reinoculation with vaccine lymph produces, in a large proportion of cases, effects local and constitutional, and a cow-pox eruption either genuine or variously modified, seems to be established by the researches of Dr. Heim; but still the *cui bono?*—the question as to the benefit derived from this revaccination arises; and it remains yet to be shown that revaccinated individuals are less susceptible of small-pox, than those who have passed only once through that operation. Further enquiries too must be made before we can agree with Heim, that the renewed susceptibility of small-pox in adult life is owing to a portion of such susceptibility being left unextinguished by the primary vaccination, however perfectly that may have succeeded, and being perhaps beyond the reach of the protective powers of the agent itself. This question perhaps, as yet, can scarcely be satisfactorily solved; although we admit that, as far as the limited experience which we have hitherto had upon the subject extends, the revaccinated are placed in the more favorable position. Of the 44,000 revaccinations within the five years over which these investigations extend, it is stated that one individual only belonging to the military class had been subsequently attacked with small-pox, and in this case the reinoculation two years before was modified, and the small-pox assumed the varioloid instead of the genuine form. This, however, though satisfactory as far as it goes, is yet imperfect, for various reasons; the houses of the infected are in Wirtemberg placed under certain restrictive or quarantine regulations, and the system of strict seclusion under which the military are kept where contagious or infectious disorders are prevalent, would render the full exposure of any of this class of persons a matter of very unfrequent occurrence. The real test, therefore, of the efficacy of revaccination must be sought amongst the civil part of the population; but of this we have no very definite details; and, indeed, the period which has elapsed since the introduction of the practice is scarcely sufficient to have afforded satisfactory results. The total number of cases of small-pox occurring within the five years is stated to have been 1677, of which 634 were genuine and 1043 varioloid; the number of inhabitants is stated at 363,298; so that 1 in 217 is the proportion of those attacked. But if this be reduced to an annual average, we shall find that the proportion is 1 in 1085; the number of the revaccinated, deducting the military, is about 30,000, and the failure of thirty of these (1 in 1000), considering that the process has not been of very early or of very easy accomplishment, would, we are inclined to think, show a not much greater amount of protection to be afforded by the second vaccination than by the primary vaccination. That there have been failures of protection to this amount among the revaccinations, or, in other words, that as many cases of small-pox have occurred in the revaccinated part of the population, we do not mean to assert; the number may have been more or fewer; but that several failures of this kind have occurred may be gathered from the reports, although it would not be a very easy matter to ascertain their precise number. The subject, however, deserves the most careful and unbiassed investigation, such as, we trust, will ere long be carried on in this country on the most extensive scale. The committee appointed at the last Anniversary of the Provincial Medical Association, for the purpose of enquiring into the present state of this country with reference to the diffusion of variola, and the protective power of vaccination, if duly seconded by the members in general, have much in their power; and we trust that they will do their utmost to execute, diligently and faithfully, the honorable and most important duty assigned to them.

There is much more in these works of Dr. Heim that we would gladly have laid before our readers. His volumes abound with matter that is of the highest interest in every point of view, whether as regards our science, or the welfare of suffering humanity; but we are compelled to pause for the present; contenting ourselves with having thus brought before the English public, for the first time, the results of much extended observation on the practice of vaccination; and concluding with the expression of our warmest approbation of the manner in which Dr. Heim has executed the very laborious and important task, to which his time and talents have been so usefully devoted.

---

